# INTENSIVE GRANDCHILD CARE AND SUCCESSFUL AGING: THE ROLE OF INTERNAL MIGRATION IN CHINA

**DOI:** 10.1093/geroni/igae098.1603

**Published:** 2024-12-31

**Authors:** Yue Hu, Helene H Fung, Neil Charness, Jason Wong, Yiqi Wang

**Affiliations:** The Chinese University of Hong Kong, Hong Kong, China (People’s Republic); The Chinese University of Hong Kong, Hong Kong, China (People’s Republic); Florida State University, Tallahassee, Florida, United States; Yale University, New Haven, Connecticut, United States; The Chinese University of Hong Kong, Hong Kong, China (People’s Republic)

## Abstract

Around two thirds of adults aged 60 years or above are providing grandchild care in China, some of whom even migrate to where their adult children live to provide grandchild care. However, it still remains unclear whether and how grandchild care provision impacts successful aging among migrant or non-migrant grandparents. This study examined whether grandchild care provision contributed to successful aging via increasing proactive coping among two groups. A questionnaire survey was conducted among 301 migrant or non-migrant grandparents who provided non-custodial grandchild care (mean age 61.2 ± 4.8, 158 female) and currently lived in a tier-1 city in China. Results showed that grandparents who provide more non-custodial care to their grandchildren experience more successful aging. Migrant grandparents are less able to rely on their proactive coping when providing intensive grandchild care as compared to non-migrant grandparents. Theoretically, this study is the first to examine the mechanism underlying the association between grandchild care provision and successful aging, contributing to the literature on positive gerontology by examining successful aging in specific contexts. Practically, findings suggest the potential guidelines to improve grandparents’ well-being. Extra support should be provided to migrant grandparents to foster their proactivity in coping with the predictive challenges of intensive grandchild care provision in terms of policy.

